# Maternal Gestational Low‐Grade Inflammation and the Risk of Anorexia Nervosa in Daughters

**DOI:** 10.1002/eat.24586

**Published:** 2025-10-24

**Authors:** Emma Saure, Pyry N. Sipilä, Cynthia M. Bulik, Elina Santti, Heljä‐Marja Surcel, Antti Latvala, Anu Raevuori

**Affiliations:** ^1^ Department of Psychiatry, Division of Adolescent Psychiatry Helsinki University Hospital Helsinki Finland; ^2^ Clinicum, Department of Public Health University of Helsinki Helsinki Finland; ^3^ Department of Psychiatry University of North Carolina at Chapel Hill Chapel Hill North Carolina USA; ^4^ Department of Medical Epidemiology and Biostatistics Karolinska Institutet Stockholm Sweden; ^5^ Department of Nutrition University of North Carolina at Chapel Hill Chapel Hill North Carolina USA; ^6^ Faculty of Medicine University of Oulu Oulu Finland; ^7^ Biobank Borealis of Northern Finland Oulu Finland; ^8^ Institute of Criminology and Legal Policy University of Helsinki Helsinki Finland; ^9^ Clinicum, Department of Psychiatry University of Helsinki Helsinki Finland

**Keywords:** anorexia nervosa, biomarkers, eating disorders, feeding and eating disorders, maternal exposure, prenatal exposure delayed effects

## Abstract

**Objective:**

Prenatal exposures have been suggested to have a programming effect on neural and metabolic development, which may affect the risk of eating disorders. We investigated the association between prospectively measured maternal gestational high‐sensitivity C‐reactive protein (hs‐CRP), an established inflammatory biomarker, and subsequent risk of AN in daughters.

**Method:**

This nested case–control study with sibling‐comparison design used systematic sampling from a register‐based cohort including all eating disorder patients in Finland born 1991–2000 and diagnosed in specialized health care. Final sample included 150 full triads of females with severe AN (ICD‐10 code F50.0), age‐ and sex‐matched population controls, and biological sister controls (total *N* = 450).

**Results:**

Mean gestational hs‐CRP values were 4.10 mg/L (SD 5.22), 4.83 mg/L (SD 4.88), and 5.53 mg/L (SD 10.36), for individuals with AN, population controls, and sister controls, respectively. Higher hs‐CRP was associated with decreased risk for AN when compared to sister controls (adjusted OR 0.68, 95% Cl 0.48–0.97, *p* = 0.03). Analyzing hs‐CRP in tertiles, maternal hs‐CRP in the highest tertile (≥ 5.13 mg/L) versus lowest tertile (≤ 1.94 mg/L) was associated with decreased risk for AN compared to sisters (adjusted OR 0.35, 95% Cl 0.15–0.80, *p* = 0.01), and to all controls combined (adjusted OR 0.52, 95% Cl 0.29–0.93, *p* = 0.03).

**Discussion:**

We found no evidence that higher gestational CRP would increase the later risk of AN. On the contrary, lower maternal low‐grade inflammation early in pregnancy was associated with an increased risk of AN in daughters.

**Summary**

- Mother's lower high‐sensitivity C‐reactive protein level in early pregnancy was associated with a higher risk of subsequent anorexia nervosa in daughters.
- Generally advantageous for metabolism and overall health, decreased low‐grade inflammation during gestation may program the offspring's metabolism towards a direction which increases the susceptibility for later anorexia nervosa in vulnerable individuals.
- Tailored recommendations for pregnant women for ensuring appropriate nutrition intake, healthy exercise, and weight gain during pregnancy could be targeted to mitigate the risk of anorexia nervosa in the next generation.

## 1 Introduction

Various exposures during the prenatal period have been suggested to have a programming effect on neural and metabolic development and may thus have a role in the development of subsequent psychiatric and neuropsychiatric disorders including anorexia nervosa (AN) in offspring (Marzola et al. 2021). One such factor, inflammation during pregnancy, has been associated with neurodevelopmental disorders, including autism and schizophrenia (Brown et al. 2014; Flinkkilä et al. 2016; Wang et al. 2025).

Maternal inflammation has been associated with higher offspring weight and fat mass both in preclinical animal models and in a human cohort study (Dahlgren et al. 2001; Gaillard et al. 2016). In a birth cohort study, self‐reported maternal infections during gestation were associated with later shape and weight concerns and increased disordered eating behavior including dieting and binge‐eating/purging behaviors among adolescent daughters (Solmi et al. 2020). Maternal exposure to rubella and chickenpox infection wave peaks was associated with a higher risk of both restrictive and binge‐purge subtypes of DSM‐IV AN in a birth cohort (Favaro et al. 2011). In addition, the mother's registered genitourinary tract infection diagnosis during pregnancy was associated with a higher risk of ICD‐10 broad and narrow AN, whereas prenatal infections were not associated with ICD‐10 broad bulimia nervosa and eating disorder not otherwise specified in two nationwide cohort studies (Larsen et al. 2021; Papini et al. 2024).

Taken together, inflammation during pregnancy may represent a pathway by which infectious and non‐infectious exposures increase the risk for psychiatric disorders. Earlier evidence suggests an association between prenatal infections and increased risk for later AN. To the best of our knowledge, the current study is the first to acquire direct evidence of the association of early gestational high‐sensitivity C‐reactive protein (hs‐CRP)—an established inflammatory biomarker—prospectively assayed in maternal sera, and the risk of AN in female offspring. In addition to index individuals with AN and population controls, we included sister controls without eating disorders, allowing us to control for factors shared by the full siblings, such as maternal and other familial characteristics, and part of the genetic predisposition for AN.

## 2 Method

### 2.1 Study Design and Population

This nested case–control study included data from the following Finnish registries: the Central Population Register, the Care Register for Health Care, and the Finnish Maternity Cohort, which is a national biobank with archived prenatal serum specimens drawn from pregnant women. Individuals in the registries are identified by a personal identification number assigned at birth or migration, which allows for linkages between the registries. From these sources, we ascertained all individuals with AN (cases), assigned female sex at birth and born between 1991 and 2000, who had a record of inpatient hospital treatment for AN (ICD‐10 code F50.0), but no other lifetime eating disorder or other ICD‐9/ICD‐10 diagnoses of mental disorders in specialized outpatient or inpatient records, and who had a biological full‐sister born since 1984 without ICD‐9/ICD‐10 diagnoses of mental disorders (eating disorder or non‐eating disorder) in the respective records (sister controls). Population controls were selected based on the date of birth (±150 days), sex, and place of birth; exclusion criteria included any ICD‐9/ICD‐10 diagnoses for eating disorders in specialized outpatient or inpatient records. Maternal gestational serum samples for each member of the triad—case, sister control, and population control—had to exist in the Finnish Maternity Cohort. Due to poor quality or too small amount of a sample in any member of the triad, 48 triads were excluded, which led to a sample of 150 females with AN, 150 sister controls, and 150 population controls.

The study was approved by the Hospital District of Southwest Finland Ethics Committee (7/2007) and the Finnish Institute of Health and Welfare (8/2016).

### 2.2 Registers

The Central Population Register includes comprehensive data on place of birth, date of death or emigration, place of residence, and biological parents.

The Care Register for Health Care is a continuation of the Finnish Hospital Discharge Register. The register identifies all recorded diagnoses for hospital admissions and recorded diagnoses for all specialized outpatient care. All Finnish citizens and permanent residents are entitled to national health insurance maintained by the state. The registry covers all mental and general hospitals, as well as all inpatient wards of local health centers, military wards, prison hospitals, and private hospitals. Primary diagnoses with up to three possible subsidiary diagnoses are included. Diagnostic information is based on clinical diagnoses made by the attending physician.

The Finnish Maternity Cohort consists of virtually all pregnancies in Finland with archived prenatal serum specimens drawn since 1983. Sera were drawn during the first or early second trimester from over 98% of pregnant women in Finland, following informed consent, for screening for HIV, syphilis, and hepatitis.

### 2.3 hs‐CRP Assay

Maternal serum samples were drawn at mean gestational week 11 (SD 3.0) (Table 1) and stored until laboratory analysis at −25°C in a protected biorepository at Biobank Borealis in Oulu, Finland. Quantitative analyses of hs‐CRP were performed using chemiluminescent microparticle immunoassays with Architect ci8200 analyzer (Abbott Diagnostics) according to the manufacturer's instructions, and by laboratory personnel blind to case–control‐sibling status. The samples were analyzed for serum total hs‐CRP mg/l (ARCHITECT 2nd Generation). Internal control samples of pooled serum were derived from pregnant women at the first trimester and were included in each set of daily assays.

**TABLE 1:** Characteristics of the study sample.

		Females with anorexia nervosa, *N* = 150	Population controls, *N* = 150	Sister controls, *N* = 150
Maternal gestational hs‐CRP level (mg/L)	Mean (SD)	4.10 (5.22)	4.83 (4.88)	5.53 (10.36)
	Range	0.26–43.96	0.28–26.46	0.23–101.19
	First tertile, *N* (≤ 1.94 mg/L)	67 (44.7%)	50 (33.3%)	54 (36.0%)
	Second tertile, *N* (1.95–5.12 mg/L)	48 (32.0%)	51 (34.0%)	48 (32.0%)
	Third tertile, *N* (≥ 5.13 mg/L)	35 (23.3%)	49 (32.7%)	48 (32.0%)
Birth year	Mean (SD)	1994 (2.5)	1994 (2.5)	1993 (4.4)
	Range	1991–2000	1991–2000	1984–2012
Sampling year of the maternal serum sample	Mean (SD)	1993 (2.6)	1993 (2.6)	1993 (4.5)
	Range	1990–2000	1990–2000	1984–2012
Sampling time (gestational week) of the maternal serum sample	Mean (SD)	11.1 (2.7)	11.8 (3.4)	10.9 (2.8)
	Range	6.4–18.9	4.3–24.1	5.1–16.6
Maternal age (years) at the expected date of delivery	Mean (SD)	29.2 (4.3)	29.9 (5.2)	29.6 (4.8)
	Range	19.0–41.1	17.8–44.0	20.0–41.9
Calcium concentration of sample (mmol/L)^a^	Mean (SD)	2.46 (0.25)	2.46 (0.31)	2.47 (0.28)
	Range	1.18–2.94	1.14–3.91	1.50–3.81
Birth order	First born *n* (%)	61 (42.0%)	47 (31.3%)	58 (39.7%)

Abbreviations: hs‐CRP, High‐sensitivity C‐reactive Protein; SD, Standard Deviation.

^a^ Calcium concentration reflects the quality of the sample.

### 2.4 Statistical Analysis

The analyses of the association between gestational hs‐CRP and AN in daughters were based on a matched case–control design using conditional logistic regression. Three comparisons were made: cases versus all controls combined, cases versus population controls, and cases versus sister controls. The conditional logistic regression takes into account the nested case–control structure of the data. In these analyses, individuals with AN were compared to their matched population controls (cases vs. population control) or to their sibling controls (cases vs. sister controls) or both combined. Due to skewed distribution, hs‐CRP values were log‐transformed before examining gestational hs‐CRP as a continuous variable in relation to the risk of AN. Hs‐CRP was also categorized into tertiles; cut‐offs were based on the values in population controls because age‐ and hs‐CRP assay timing–matched whole female population distribution of hs‐CRP/CRP were not available (Brown et al. 2014). Analyses were adjusted for pregnancy weeks at serum sample collection, calcium concentration of the sample, and birth order (first born vs. others). If values applied in the adjustment were missing, the pair or triad with an individual with a missing value was not included in adjusted analysis. Birth order between the groups was assessed using the *χ* ^2^ test. To assess whether exclusion of individuals with missing data in the adjusted analysis explains the potential differences between the adjusted and unadjusted results, we conducted the unadjusted analysis also by excluding triads with missing values. We conducted three additional sensitivity analyses with alternative adjustments: (1) replacing birth order with birth year, (2) replacing birth order with maternal age, and (3) additionally adjusting for maternal smoking during pregnancy (yes/no), hypertensive disorders of pregnancy (yes/no), and gestational diabetes (yes/no). Statistical analyses were performed using Stata, version 18.

## 3 Results

### 3.1 Background Characteristics

Background characteristics are presented in Table 1. Individuals with AN tended to be first born more often than sister or population controls (*X* ^2^ = 3.6, *p* = 0.17, Table 1). Of those with AN who were first born, 19.3% (*n* = 29) had hs‐CRP in the lowest tertile, whereas the same was true for 16.2% (*n* = 24) and 14.7% (*n* = 22) of sister and population controls, respectively (*X* ^2^ = 0.10, *p* = 0.95). Of population controls, 20 (13.3%) had one or more recorded (non‐eating disorder) mental disorder diagnoses, most commonly depressive disorder (*N* = 7, 4.7%), followed by anxiety disorders including obsessive compulsive disorder, reactions to stress and adjustment disorders, and developmental disorders of speech and language. The mean age at the first AN diagnosis among cases was 14.6 years (SD 2.6 years).

### 3.2 Maternal hs‐CRP and Later Risk of AN in Daughters

Modeled as a continuous log‐transformed variable, higher maternal hs‐CRP was associated with a lower risk of AN compared to sister controls (Table 2). Comparison to all controls combined yielded a comparable, nonsignificant association. Analyzing hs‐CRP in tertiles, maternal hs‐CRP in the highest tertile versus the lowest tertile was associated with a decreased risk for AN compared to all controls and to sister controls. Respective comparison to population controls alone yielded no association (Table 2). The findings remained virtually unchanged in a sensitivity analysis excluding triads with missing values from unadjusted analyses (Table S1) as well as in sensitivity analyses controlling for birth year instead of birth order, maternal age instead of birth order, and maternal smoking, hypertensive disorder, and gestational diabetes (Tables S2–S4).

**TABLE 2:** The association of prenatal high‐sensitivity C‐reactive Protein CRP (hs‐CRP) with anorexia nervosa among daughters by hs‐CRP as a log‐transformed continuous variable and by tertiles.

	Females with anorexia nervosa versus all controls combined		Females with anorexia nervosa versus population controls		Females with anorexia nervosa versus sister controls	
	OR (95% Cl)	*p*	OR (95% Cl)	*p*	OR (95% Cl)	*p*
hs‐CRP, continuous						
Unadjusted	0.78 (0.62–0.97)	0.02	0.79 (0.62–0.99)	0.04	0.73 (0.54–0.99)	0.04
Adjusted^a^	0.80 (0.63–1.02)	0.07	0.86 (0.66–1.10)	0.23	0.68 (0.48–0.97)	0.03
hs‐CRP, first tertile (≤ 1.94 mg/L), reference						
	1.00		1.00		1.00	
hs‐CRP, second tertile (1.95–5.12 mg/L)						
Unadjusted	0.70 (0.43–1.12)	0.14	0.69 (0.41–1.17)	0.17	0.70 (0.36–1.34)	0.28
Adjusted^a^	0.71 (0.43–1.17)	0.18	0.74 (0.43–1.27)	0.27	0.62 (0.31–1.25)	0.18
hs‐CRP, third tertile (≥ 5.13 mg/L)						
Unadjusted	0.49 (0.28–0.85)	0.01	0.54 (0.30–0.95)	0.03	0.40 (0.18–0.86)	0.02
Adjusted^a^	0.52 (0.29–0.93)	0.03	0.65 (0.35–1.21)	0.17	0.35 (0.15–0.80)	0.01

Abbreviations: CI, Confidence Interval; OR, Odds Ratio.

^a^ Adjusted for calcium concentration of the sample, pregnancy weeks at the time of the collection of the serum sample, and the birth order (first born vs. others). If values applied in the adjustment were missing in any member of the pair/triad (calcium concentration, missing *n* = 6; first born versus others, missing *n* = 2), the pair/triad was not included in the adjusted analysis.

## 4 Discussion

Contrary to earlier indirect evidence showing links between prenatal infections and increased risk for later AN and disordered eating behavior, we found no evidence of an association between directly and prospectively assessed increased maternal low‐grade inflammation (hs‐CRP) and elevated risk for AN in daughters. Furthermore, when females with AN were compared to all controls combined or healthy sisters only, we observed an inverse association between maternal low‐grade inflammation and offspring AN, such that lower maternal hs‐CRP was associated with an increased risk of AN in daughters. This suggests that decreased maternal low‐grade inflammation, although normally indicating improved physiological homeostasis and metabolically optimal prenatal milieu, may have a role in the later development of AN.

Our findings align with reports indicating that AN shares a genetic basis with lower levels of unfavorable anthropometric and metabolic markers (Duncan et al. 2017; Hübel et al. 2019; Watson et al. 2019). Genome‐wide association studies have shown negative associations between AN and an array of anthropometric and metabolic traits, including body mass index, fat mass, obesity, type‐2 diabetes, LDL cholesterol, leptin, and insulin‐related traits (Hübel et al. 2019; Watson et al. 2019). Many of these traits, when shifting towards a metabolically unfavorable direction, are involved in the pathogenesis of non‐communicable chronic inflammatory diseases (Rohm et al. 2022), other psychiatric disorders (Hübel et al. 2019), and are also linked to increased low‐grade inflammation (Hübel et al. 2019; Rohm et al. 2022). Lowered low‐grade inflammation during gestation that is generally advantageous for metabolism and overall mental and physical health in both mother and offspring may, however, have potential for programming the offspring's metabolism towards a direction which increases the susceptibility for later AN in a group of vulnerable individuals. This is supported by our findings where eating disorder‐free sister controls with the same mothers as females with AN showed the highest mean hs‐CRP levels during gestation, also relative to population controls. This suggests a protective effect of a relatively higher level of low‐grade inflammation among females with increased familial risk for AN. It is also possible that lower low‐grade inflammation serves as a marker of the mother's restrictive eating during that specific pregnancy, which may, in turn, increase the risk of later AN in daughters through metabolic programming.

### 4.1 Strengths and Limitations

Strengths of this study include direct assessment of hs‐CRP levels from prospectively collected maternal serum samples. The inclusion of sister controls allowed controlling for factors shared by the siblings, such as time‐invariant socioeconomic influences and part of genetic effects (Frisell 2021). In the Finnish Maternity Cohort, CRP and hs‐CRP analyses have been run from thousands of samples with varying time between sampling and laboratory analysis. In this study, hs‐CRP levels were analyzed mean 22.5 (SD 3.3) years after sampling; those analyzed up to 30 years afterwards have shown no biodegradation (unpublished data), suggesting that hs‐CRP is well‐preserved (Chudal et al. 2020). Moreover, the equal time between case and control sampling and hs‐CRP analyses eliminates potential effects of the storage. Participants were drawn systematically from national registries to minimize selection bias. Sampling of cases covered merely individuals with severe, restricting subtype AN. Differentiation of subtypes is essential due to potential etiological differences, as binge‐eating/purging subtype AN may have closer proximity with eating disorders characterized by binge eating, which share genomic variants with overweight and obesity (Bulik et al. 2022); these, in turn, are associated with increased low‐grade inflammation (Moreto et al. 2013). Despite nationwide sampling, the number of participants was relatively low. Results may have been more robust in a larger sample, and we cannot exclude type II error. Limitations also include examination of low‐grade inflammation with a single marker at a single time point in early pregnancy. The availability of a range of inflammatory biomarkers throughout pregnancy could have enabled examination of more specific inflammatory markers in different stages of pregnancy. We relied solely on register‐based diagnostic information and lacked data concerning maternal weight, body mass index, and eating disorders. We did not exclude participants with elevated hs‐CRP values (> 20 mg/L), which were observed in 13 individuals (3 with AN, 5 sister controls, and 5 population controls). Among sister controls, two markedly elevated values were observed (over 60 mg/L). Nonetheless, our findings indicate that *lower* CRP may increase the risk for AN, whereas *higher* CRP appears protective, a pattern further supported by the wider distribution of maternal CRP values in pregnancies without subsequent AN.

### 4.2 Conclusions and Future Directions

This study suggests an association between decreased maternal low‐grade inflammation during early pregnancy—which generally indicates a metabolically healthier prenatal milieu—and a later increased risk of severe AN among daughters. The present findings imply that prenatal conditions normally considered optimal and to be pursued could have a different meaning in females with increased vulnerability for developing AN. Interestingly, the findings are parallel with the evidence suggesting a shared genetic basis of AN and lower levels of unfavorable metabolic markers. Future studies with prospective biobank data in large patient cohorts combining genome‐wide association and plasma proteomics data with information about maternal eating disorders and comorbidities could be a step forward to characterize the role of inflammatory biomarkers in the risk of AN. By genetically informed characterization of the maternal metabolic risk profiles, tailored recommendations for ensuring adequate and appropriate nutrition intake, healthy exercise, and weight gain during pregnancy could be targeted to mitigate the risk of this life‐threatening eating disorder in the next generation.

## Author Contributions

**Emma Saure:** investigation, visualization, writing – original draft, writing – review and editing, methodology. **Pyry N. Sipilä:** methodology, investigation, visualization, writing – review and editing, supervision, writing – original draft. **Cynthia M. Bulik:** investigation, visualization, writing – original draft, writing – review and editing. **Elina Santti:** project administration, writing – review and editing, investigation, visualization. **Heljä‐Marja Surcel:** conceptualization, investigation, methodology, visualization, project administration, writing – review and editing. **Antti Latvala:** investigation, visualization, writing – review and editing, supervision. **Anu Raevuori:** conceptualization, methodology, supervision, writing – original draft, writing – review and editing, project administration, visualization, investigation.

## Conflicts of Interest

A.R. holds equity ownership in Meru Health Inc. outside the submitted work. C.M.B. receives royalties from Person Education Inc. and has served as a consultant to Orbimed. The other authors declare no conflicts of interest.

## Supporting information


**Table S1:** The association of prenatal high‐sensitivity C‐reactive Protein (hs‐CRP) with anorexia nervosa among daughters by hs‐CRP as a log‐transformed continuous variable and by tertiles. In this table, unadjusted analysis includes only those pairs or triads without any missing values. Adjusted analysis are adjusted for calcium concentration of the sample, pregnancy weeks at the time of the collection of the serum sample, and the birth order (first born vs. others).
**Table S2:** The association of prenatal high‐sensitivity C‐reactive Protein CRP (hs‐CRP) with anorexia nervosa among daughters by hs‐CRP as a log‐transformed continuous variable and by tertiles. Adjusted analysis are adjusted for calcium concentration of the sample, pregnancy weeks at the time of the collection of the serum sample, and the birth year.
**Table S3:** The association of prenatal high‐sensitivity C‐reactive Protein CRP (hs‐CRP) with anorexia nervosa among daughters by hs‐CRP as a log‐transformed continuous variable and by tertiles. Adjusted analysis are adjusted for calcium concentration of the sample, pregnancy weeks at the time of the collection of the serum sample, and maternal age at the expected date of delivery.
**Table S4:** The association of prenatal high‐sensitivity C‐reactive Protein CRP (hs‐CRP) with anorexia nervosa among daughters by hs‐CRP as a log‐transformed continuous variable and by tertiles. Adjusted analysis are adjusted for calcium concentration of the sample, the birth order (first born vs. others), maternal smoking during pregnancy, hypertensive disorders of pregnancy, and gestational diabetes.

## Data Availability

The data that support the findings of this study are available from the corresponding author upon reasonable request. The data are not publicly available due to privacy or ethical restrictions.
