# Supplementary material for: Maternal Gestational Low‐Grade Inflammation and the Risk of Anorexia Nervosa in Daughters
Source: Int J Eat Disord. 2025 Oct 24;59(2):401–6. doi: 10.1002/eat.24586 (PMC12884243; doi:10.1002/eat.24586)
Supplement: Supplementary file 1 — Table S1: The association of prenatal high‐sensitivity C‐reactive Protein (hs‐CRP) with anorexia nervosa among daughters by hs‐CRP as a log‐transformed continuous variable and by tertiles. In this table, unadjusted analysis includes only those pairs or triads without any missing values. Adjusted analysis are adjusted for calcium concentration of the sample, pregnancy weeks at the time of the collection of the serum sample, and the birth order (first born vs. others). Table S2: The association of prenatal high‐sensitivity C‐reactive Protein CRP (hs‐CRP) with anorexia nervosa among daughters by hs‐CRP as a log‐transformed continuous variable and by tertiles. Adjusted analysis are adjusted for calcium concentration of the sample, pregnancy weeks at the time of the collection of the serum sample, and the birth year. Table S3: The association of prenatal high‐sensitivity C‐reactive Protein CRP (hs‐CRP) with anorexia nervosa among daughters by hs‐CRP as a log‐transformed continuous variable and by tertiles. Adjusted analysis are adjusted for calcium concentration of the sample, pregnancy weeks at the time of the collection of the serum sample, and maternal age at the expected date of delivery. Table S4: The association of prenatal high‐sensitivity C‐reactive Protein CRP (hs‐CRP) with anorexia nervosa among daughters by hs‐CRP as a log‐transformed continuous variable and by tertiles. Adjusted analysis are adjusted for calcium concentration of the sample, the birth order (first born vs. others), maternal smoking during pregnancy, hypertensive disorders of pregnancy, and gestational diabetes. [file EAT-59-401-s001.docx]

**Supplementary table 1.** The association of prenatal high-sensitivity C-reactive Protein (hs-CRP) with anorexia nervosa among daughters by hs-CRP as a log-transformed continuous variable and by tertiles. In this table, unadjusted analysis includes only those pairs or triads without any missing values. Adjusted analysis are adjusted for calcium concentration of the sample, pregnancy weeks at the time of the collection of the serum sample, and the birth order (first born *versus* others).

Abbreviations: Odds Ratio, OR; Confidence Interval, CI

|  | | **Females with anorexia nervosa *versus***  **all controls combined** | | **Females with anorexia nervosa *versus* population controls** | | **Females with anorexia nervosa *versus***  **sister controls** | |
| --- | --- | --- | --- | --- | --- | --- | --- |
|  | | **OR**  **(95 % Cl)** | **P** | **OR**  **(95 % Cl)** | **P** | **OR**  **(95 % Cl)** | **P** |
| **hs-CRP, continuous** | **Unadjusted** | 0.78  (0.63–0.98) | 0.03 | 0.78  (0.62–0.99) | 0.04 | 0.74  (0.54­–1.01) | 0.05 |
|  | **Adjusted** | 0.80  (0.63–1.02) | 0.07 | 0.86  (0.66–1.10) | 0.23 | 0.68  (0.48–0.97) | 0.03 |
| **hs-CRP, first tertile**  **(**≤ **1.94 mg/l), reference** | | 1.00 |  | 1.00 |  | 1.00 |  |
| **hs-CRP, second tertile**  **(1.95–5.12 mg/l)** | **Unadjusted** | 0.70  (0.43–1.14) | 0.14 | 0.68  (0.40–1.16) | 0.16 | 0.68  (0.35–1.33) | 0.26 |
|  | **Adjusted** | 0.71  (0.43–1.17) | 0.18 | 0.74  (0.43–1.27) | 0.27 | 0.62  (0.31–1.25) | 0.18 |
| **hs-CRP,**  **third tertile**  **(≥ 5.13 mg/l)** | **Unadjusted** | 0.49  (0.28-0.86) | 0.01 | 0.53  (0.29–0.94) | 0.03 | 0.39  (0.18–0.86) | 0.02 |
|  | **Adjusted** | 0.52  (0.29–0.93) | 0.03 | 0.65  (0.35–1.21) | 0.17 | 0.35  (0.15–0.80) | 0.01 |

**Supplementary table 2**. The association of prenatal high-sensitivity C-reactive Protein CRP (hs-CRP) with anorexia nervosa among daughters by hs-CRP as a log-transformed continuous variable and by tertiles. Adjusted analysis are adjusted for calcium concentration of the sample, pregnancy weeks at the time of the collection of the serum sample, and the birth year.

Abbreviations: Odds Ratio, OR; Confidence Interval, CI

|  | | **Females with anorexia nervosa *versus***  **all controls combined** | | **Females with anorexia nervosa *versus* population controls** | | **Females with anorexia nervosa *versus***  **sister controls** | |
| --- | --- | --- | --- | --- | --- | --- | --- |
|  | | **OR**  **(95 % Cl)** | **P** | **OR**  **(95 % Cl)** | **P** | **OR**  **(95 % Cl)** | **P** |
| **hs-CRP, continuous** | **Unadjusted** | 0.78  (0.62–0.97) | 0.02 | 0.79  (0.62–0.99) | 0.04 | 0.73  (0.54­–0.99) | 0.04 |
|  | **Adjusted** | 0.80  (0.63–1.00) | 0.05 | 0.83  (0.65–1.06) | 0.14 | 0.72  (0.52–1.01) | 0.06 |
| **hs-CRP, first tertile**  **(**≤ **1.94 mg/l), reference** | | 1.00 |  | 1.00 |  | 1.00 |  |
| **hs-CRP, second tertile**  **(1.95–5.12 mg/l)** | **Unadjusted** | 0.70  (0.43–1.12) | 0.14 | 0.69  (0.41–1.17) | 0.17 | 0.70  (0.36–1.34) | 0.28 |
|  | **Adjusted** | 0.71  (0.43–1.15) | 0.16 | 0.72  (0.42–1.24) | 0.24 | 0.65  (0.33–1.30) | 0.23 |
| **hs-CRP,**  **third tertile**  **(≥ 5.13 mg/l)** | **Unadjusted** | 0.49  (0.28-0.85) | 0.01 | 0.54  (0.30–0.95) | 0.03 | 0.40  (0.18–0.86) | 0.02 |
|  | **Adjusted** | 0.51  (0.28–0.91) | 0.02 | 0.72  (0.33–1.11) | 0.10 | 0.39  (0.17–0.87) | 0.02 |

**Supplementary table 3**. The association of prenatal high-sensitivity C-reactive Protein CRP (hs-CRP) with anorexia nervosa among daughters by hs-CRP as a log-transformed continuous variable and by tertiles. Adjusted analysis are adjusted for calcium concentration of the sample, pregnancy weeks at the time of the collection of the serum sample, and maternal age at the expected date of delivery

Abbreviations: Odds Ratio, OR; Confidence Interval, CI

|  | | **Females with anorexia nervosa *versus***  **all controls combined** | | **Females with anorexia nervosa *versus* population controls** | | **Females with anorexia nervosa *versus***  **sister controls** | |
| --- | --- | --- | --- | --- | --- | --- | --- |
|  | | **OR**  **(95 % Cl)** | **P** | **OR**  **(95 % Cl)** | **P** | **OR**  **(95 % Cl)** | **P** |
| **hs-CRP, continuous** | **Unadjusted** | 0.78  (0.62–0.97) | 0.02 | 0.79  (0.62–0.99) | 0.04 | 0.73  (0.54­–0.99) | 0.04 |
|  | **Adjusted** | 0.80  (0.63–1.01) | 0.06 | 0.85  (0.66–1.09) | 0.19 | 0.71  (0.51–0.99) | 0.05 |
| **hs-CRP, first tertile**  **(**≤ **1.94 mg/l), reference** | | 1.00 |  | 1.00 |  | 1.00 |  |
| **hs-CRP, second tertile**  **(1.95–5.12 mg/l)** | **Unadjusted** | 0.70  (0.43–1.12) | 0.14 | 0.69  (0.41–1.17) | 0.17 | 0.70  (0.36–1.34) | 0.28 |
|  | **Adjusted** | 0.73  (0.44–1.19) | 0.20 | 0.76  (0.44–1.30) | 0.32 | 0.65  (0.32–1.30) | 0.22 |
| **hs-CRP,**  **third tertile**  **(≥ 5.13 mg/l)** | **Unadjusted** | 0.49  (0.28-0.85) | 0.01 | 0.54  (0.30–0.95) | 0.03 | 0.40  (0.18–0.86) | 0.02 |
|  | **Adjusted** | 0.51  (0.28–0.91) | 0.02 | 0.63  (0.34–1.69) | 0.14 | 0.38  (0.17-0.85) | 0.02 |

**Supplementary table 4**. The association of prenatal high-sensitivity C-reactive Protein CRP (hs-CRP) with anorexia nervosa among daughters by hs-CRP as a log-transformed continuous variable and by tertiles. Adjusted analysis are adjusted for calcium concentration of the sample, the birth order (first born *versus* others), maternal smoking during pregnancy, hypertensive disorders of pregnancy, and gestational diabetes.

Abbreviations: Odds Ratio, OR; Confidence Interval, CI

|  | | **Females with anorexia nervosa *versus***  **all controls combined** | | **Females with anorexia nervosa *versus* population controls** | | **Females with anorexia nervosa *versus***  **sister controls** | |
| --- | --- | --- | --- | --- | --- | --- | --- |
|  | | **OR**  **(95 % Cl)** | **P** | **OR**  **(95 % Cl)** | **P** | **OR**  **(95 % Cl)** | **P** |
| **hs-CRP, continuous** | **Unadjusted** | 0.78  (0.62–0.97) | 0.02 | 0.79  (0.62–0.99) | 0.04 | 0.73  (0.54­–0.99) | 0.04 |
|  | **Adjusted** | 0.82  (0.64–1.05) | 0.12 | 0.92  (0.70–1.21) | 0.54 | 0.64  (0.43–0.94) | 0.02 |
| **hs-CRP, first tertile**  **(**≤ **1.94 mg/l), reference** | | 1.00 |  | 1.00 |  | 1.00 |  |
| **hs-CRP, second tertile**  **(1.95–5.12 mg/l)** | **Unadjusted** | 0.70  (0.43–1.12) | 0.14 | 0.69  (0.41–1.17) | 0.17 | 0.70  (0.36–1.34) | 0.28 |
|  | **Adjusted** | 0.79  (0.47–1.33) | 0.37 | 0.86  (0.49–1.52) | 0.61 | 0.67  (0.31–1.43) | 0.30 |
| **hs-CRP,**  **third tertile**  **(≥ 5.13 mg/l)** | **Unadjusted** | 0.49  (0.28-0.85) | 0.01 | 0.54  (0.30–0.95) | 0.03 | 0.40  (0.18–0.86) | 0.02 |
|  | **Adjusted** | 0.55  (0.30–1.02) | 0.06 | 0.74  (0.39–1.43) | 0.37 | 0.36  (0.15–0.88) | 0.03 |
